# Supplementary material for: A positive statistical benchmark to assess network agreement
Source: Nat Commun. 2023 May 24;14:2988. doi: 10.1038/s41467-023-38625-z (PMC10209207; doi:10.1038/s41467-023-38625-z)
Supplement: Supplementary file 1 — Supplementary Information [file 41467_2023_38625_MOESM1_ESM.pdf]

A positive statistical benchmark to assess network agreement
Supplementary Information

Bingjie Hao[1] **and István A. Kovács**[1,2,†]
[1]Department of Physics and Astronomy, Northwestern University, Evanston, IL 60208
[2]Northwestern Institute on Complex Systems, Northwestern University, Evanston, IL 60208
[†]istvan.kovacs@northwestern.edu

Contents

1 Supplement Tables **2**
2 Supplement Figures **3**
Supplementary References **3**

1 Supplement Tables

Table S1. A summary of yeast datasets.

dataset	nodes	links	density
YeRI	1,346	1,880	0.002077
Ito-core	766	738	0.002519
Uetz-screen	747	607	0.002179
CCSB-YI1	1,206	1,605	0.002209
Tarassov	1,078	2,534	0.004365
Sys-NB-06	3,067	12,968	0.002758
I3D	1,078	1,761	0.003034
AF+RF	1,280	1,106	0.001351
Lit-BM-20	2,666	5,056	0.001423
BioGRID	5,992	130,999	0.007298
STRING(HsC)	2,550	29,600	0.009108
STRING(HC)	2,974	16,823	0.003805
STRING(MC)	4,705	39,072	0.003531
STRING(LC)	5,944	154,885	0.008769
PRS	149	108	0.009795
RRS	373	198	0.002854
co-complex	2,317	17,864	0.006658
co-expression	1,742	5,000	0.003297
co-localization	702	799	0.003247
co-annotation	3,955	509,499	0.065161
GI-PSN	4,770	34,109	0.002999
GI_ExE_pos	849	29,281	0.081342
GI_ExE_neg	855	42,933	0.117597
GI_NxN_pos	4,676	149,808	0.013706
GI_NxN_neg	4,682	224,748	0.02051

Table S2. A summary of human datasets.

dataset	nodes	links	density
HuRI	8,245	52,067	0.001532
HI-I-05	1,500	2,561	0.002278
Venkatesan-09	195	187	0.009886
HI-II-14	4,111	13,118	0.001553
Yu-11	1,133	1,127	0.001757
I3D-H	3,155	4,354	0.000875
BioPlex	12,408	97,078	0.001261
Qubic	4,927	23,848	0.001965
Lit-BM-17	5,956	12,758	0.000719
STRING(HsC)-H	6,662	39,567	0.001783
HuRI-PSN	4,229	33,125	0.003705

2 Supplement Figures

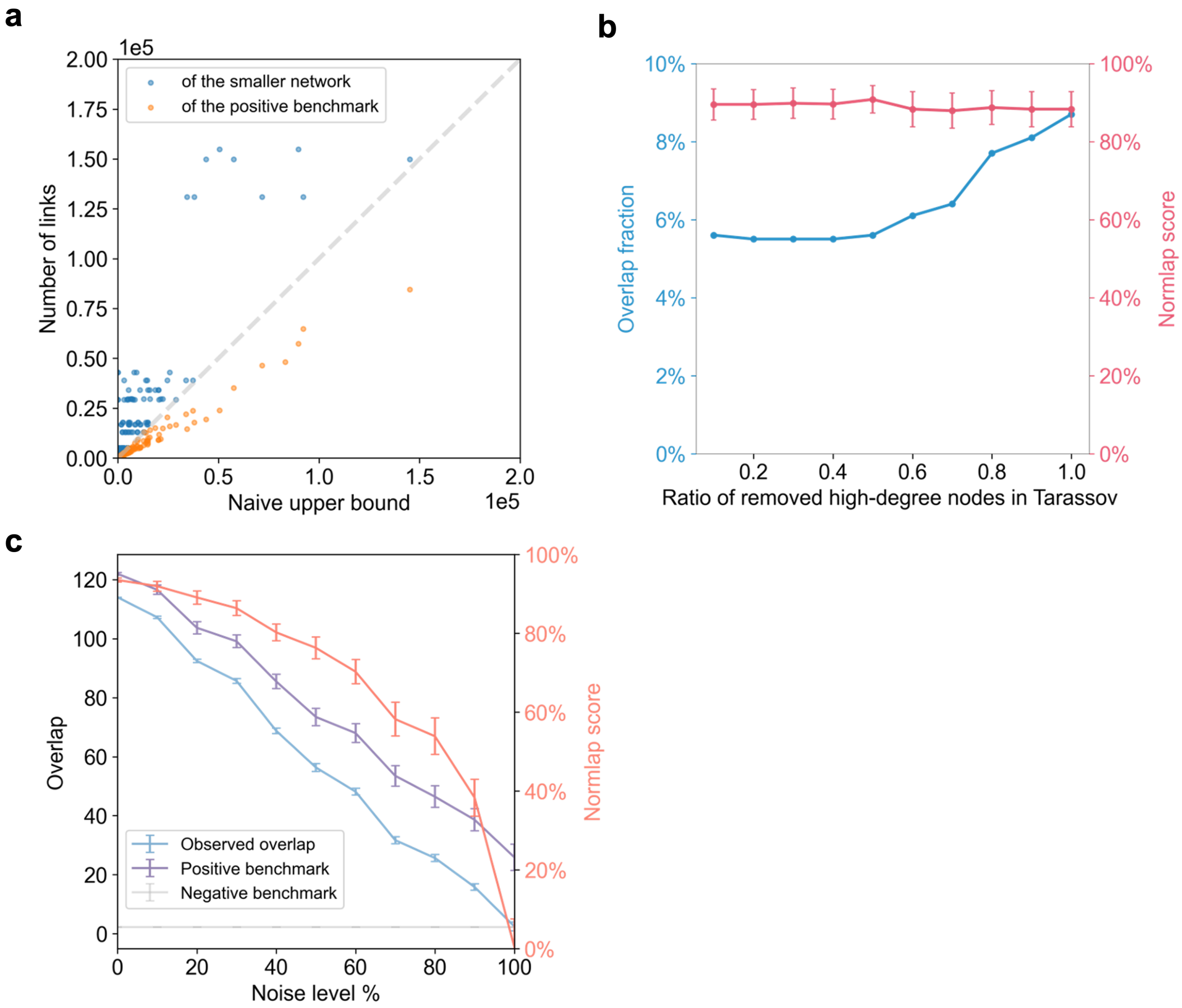

Figure S1. (a) The comparison between the naive upper bound and the number of links of the smaller network and the positive benchmark. (b) The Normlap score dependency on the removal of high-degree nodes in Tarassov[1] compared to I3D[2]. As we randomly remove high-degree nodes from Tarassov, the overlap fraction gradually increases while the Normlap score remains consistent. (c) Non-linearity of the Normlap score. The CCSB-YI1[3] is partially randomized according to the noise level while the degree sequence is preserved. The plot showed a comparison with the YeRI[4] network. Data are presented as mean $\pm$ SD in (b),(c).

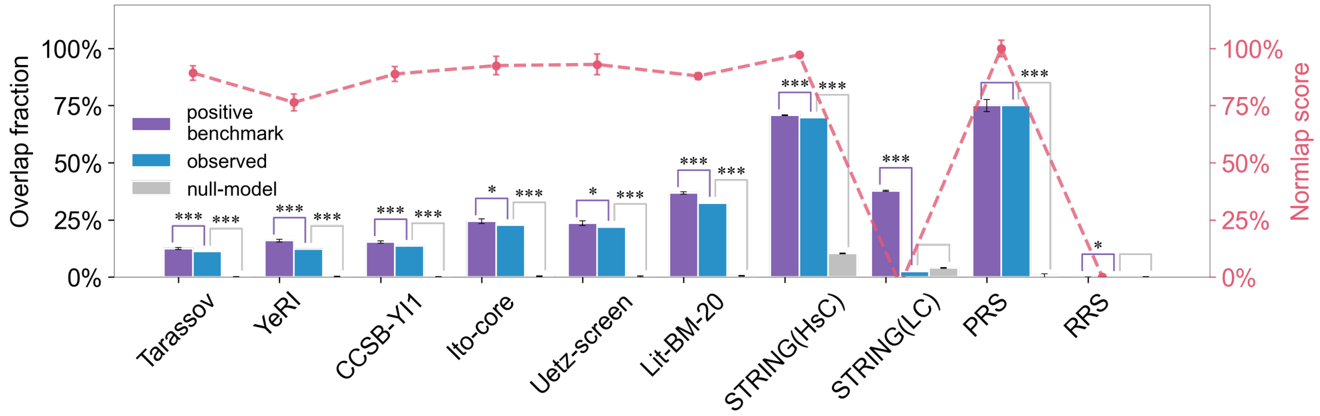

Figure S2. Overlap fraction of various yeast PPI networks compared to co-complex network[5,6]. The proposed positive benchmark is shown in purple, leading to the normalized overlap (Normlap) score in red. In gray, we show a degree-preserved randomized co-complex network as a null model for reference. The significance of the observed overlap compared to the null model or the positive benchmark is determined by a one-sided p-value (see Methods) and indicated by *: $p < 0.05$, **: $p < 0.005$, ***: $p < 0.0005$. Note that the benchmark, Normlap score and related standard deviation are directly calculated instead of being averaged across random samples, as described in the Methods section. Data are presented as mean $\pm$ SD.

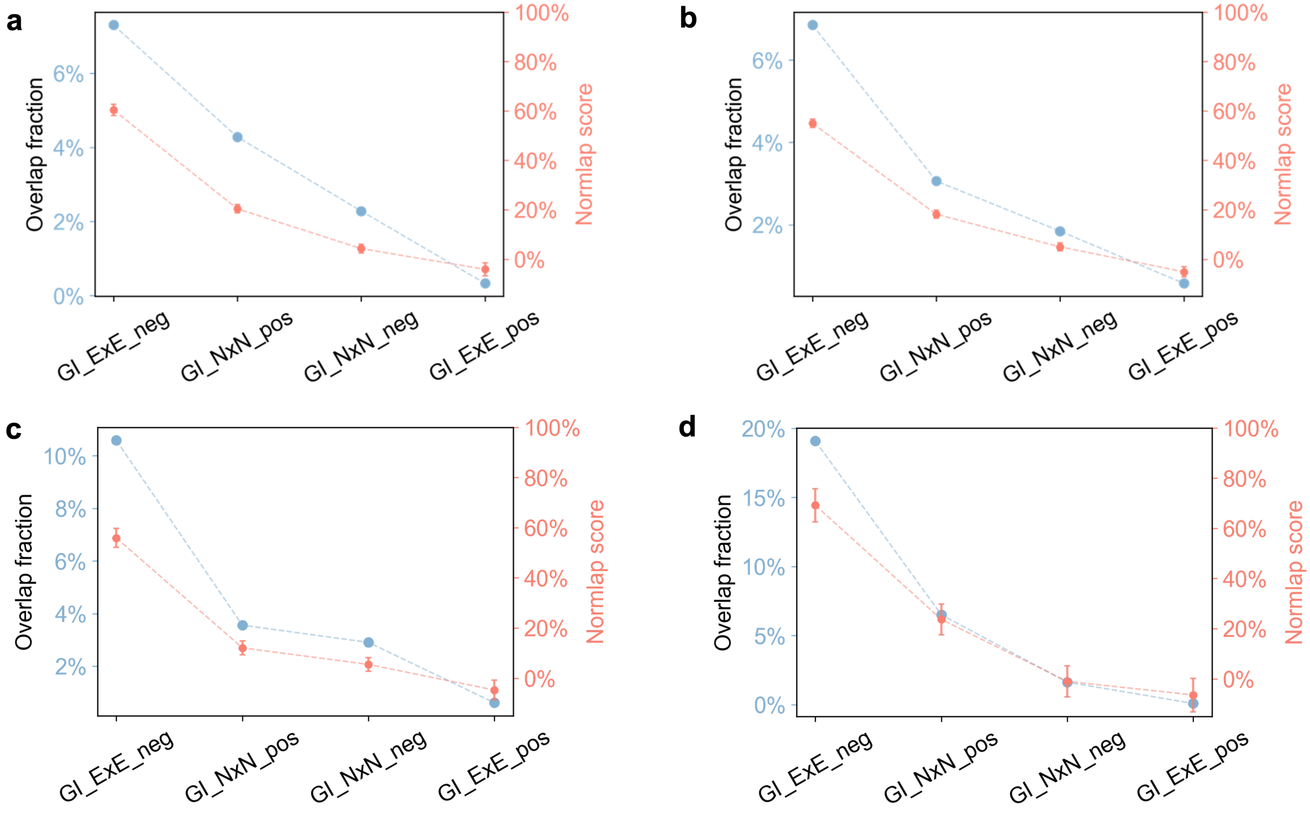

Figure S3. Different aspects of yeast GI network compared with yeast (a) co-complex network. (b) STRING(HsC) network. (c) Lit-BM-20 network. (d) AF+RF network. Data are presented as mean $\pm$ SD.

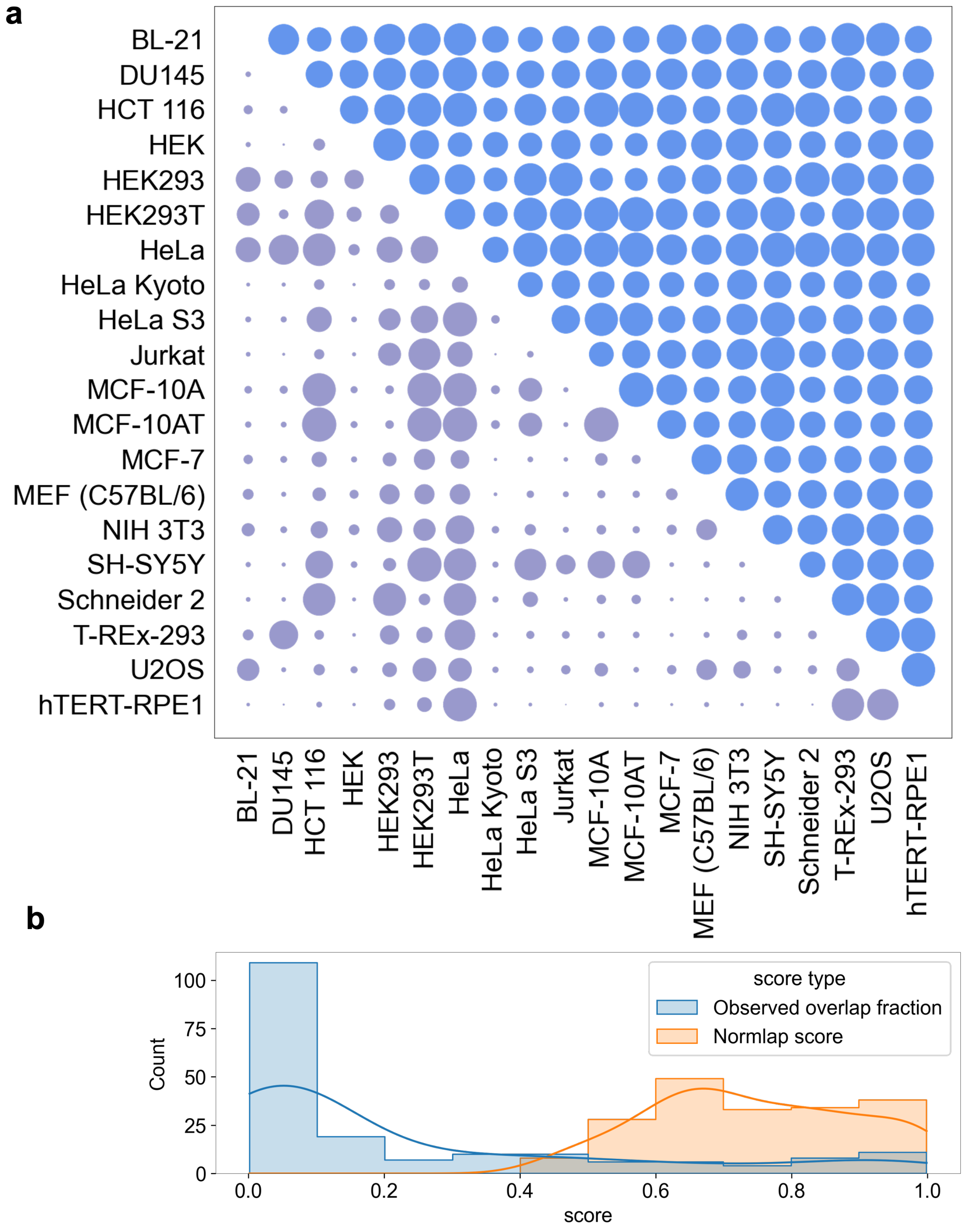

Figure S4. The comparison between human PPI networks mapped from different cells. (a) Normlap score (upper triangle) and observed overlap fraction (lower triangle) between human PPI networks mapped from different cells. (b) The distribution of observed overlap fraction and Normlap score. The Normlap score shows a more uniform picture than the Observed overlap fraction.

Supplementary References

1. Tarassov, K. *et al.* An in Vivo Map of the Yeast Protein Interactome. *Science* **320**, 1465–1470 (2008).

2. Mosca, R., Céol, A. & Aloy, P. Interactome3D: Adding structural details to protein networks. *Nat. Methods* **10**, 47–53 (2013).

3. Yu, H. *et al.* High-quality binary protein interaction map of the yeast interactome network. *Science* **322**, 104–110 (2008).

4. Lambourne, L. *et al.* Binary Interactome Models of Inner- Versus Outer-Complexome Organization. Preprint at https://www.biorxiv.org/content/10.1101/2021.03.16.435663v3. (2021).

5. Baryshnikova, A. *et al.* Quantitative analysis of fitness and genetic interactions in yeast on a genome scale. *Nat. Methods* **7**, 1017–1024 (2010).

6. Benschop, J. J. *et al.* A Consensus of Core Protein Complex Compositions for Saccharomyces cerevisiae. *Mol. Cell* **38**, 916–928 (2010).
