# Supplementary material for: A positive statistical benchmark to assess network agreement
Source: Nat Commun. 2023 May 24;14:2988. doi: 10.1038/s41467-023-38625-z (PMC10209207; doi:10.1038/s41467-023-38625-z)
Supplement: Supplementary file 3 — Reporting Summary [file 41467_2023_38625_MOESM3_ESM.pdf]

nature portfolio

Corresponding author(s): István Kovács

Last updated by author(s): Apr 28, 2023

Reporting Summary

Nature Portfolio wishes to improve the reproducibility of the work that we publish. This form provides structure for consistency and transparency in reporting. For further information on Nature Portfolio policies, see our Editorial Policies and the Editorial Policy Checklist.

Statistics

For all statistical analyses, confirm that the following items are present in the figure legend, table legend, main text, or Methods section.

n/a	Confirmed	
☒	☐	The exact sample size (*n*) for each experimental group/condition, given as a discrete number and unit of measurement
☒	☐	A statement on whether measurements were taken from distinct samples or whether the same sample was measured repeatedly
☐	☒	The statistical test(s) used AND whether they are one- or two-sided<br>*Only common tests should be described solely by name; describe more complex techniques in the Methods section.*
☒	☐	A description of all covariates tested
☐	☒	A description of any assumptions or corrections, such as tests of normality and adjustment for multiple comparisons
☐	☒	A full description of the statistical parameters including central tendency (e.g. means) or other basic estimates (e.g. regression coefficient) AND variation (e.g. standard deviation) or associated estimates of uncertainty (e.g. confidence intervals)
☐	☒	For null hypothesis testing, the test statistic (e.g. *F*, *t*, *r*) with confidence intervals, effect sizes, degrees of freedom and *P* value noted<br>*Give P values as exact values whenever suitable.*
☒	☐	For Bayesian analysis, information on the choice of priors and Markov chain Monte Carlo settings
☒	☐	For hierarchical and complex designs, identification of the appropriate level for tests and full reporting of outcomes
☐	☒	Estimates of effect sizes (e.g. Cohen's *d*, Pearson's *r*), indicating how they were calculated

Our web collection on statistics for biologists contains articles on many of the points above.

Software and code

Policy information about availability of computer code

Data collection	All related data are obtained from the references listed in the Method section. Additional data was used from the following public databases:<br>BioGRID v4.4.210<br>STRING v11.5<br>No software was used to collect the data.
Data analysis	The data was analyzed with python 3.10.6. The operating system is MacOS Ventura (M1 chip). Analysis was performed by numpy1.18.1, pandas 1.0.0 and scipy 1.4.1. The custom algorithms and code for repeating the analysis are provided at https://github.com/hbj153/normlap_paper.

For manuscripts utilizing custom algorithms or software that are central to the research but not yet described in published literature, software must be made available to editors and reviewers. We strongly encourage code deposition in a community repository (e.g. GitHub). See the Nature Portfolio guidelines for submitting code & software for further information.

Data

Policy information about availability of data

All manuscripts must include a data availability statement. This statement should provide the following information, where applicable:

- Accession codes, unique identifiers, or web links for publicly available datasets
- A description of any restrictions on data availability
- For clinical datasets or third party data, please ensure that the statement adheres to our policy

All raw data resources used are publicly available. They are processed as described in the Methods section. The processed data used for analysis in this article are available at: https://github.com/hbj153/normlap_paper or in Zenodo database under accession code 7872611. Source data are provided with this paper.
Large scale database data can be downloaded from BioGRID and STRING databases.

Human research participants

Policy information about studies involving human research participants and Sex and Gender in Research.

Reporting on sex and gender	This is not relevant to our study.
Population characteristics	This is not relevant to our study.
Recruitment	This is not relevant to our study.
Ethics oversight	This is not relevant to our study.

Note that full information on the approval of the study protocol must also be provided in the manuscript.

Field-specific reporting

Please select the one below that is the best fit for your research. If you are not sure, read the appropriate sections before making your selection.

☒ Life sciences ☐ Behavioural & social sciences ☐ Ecological, evolutionary & environmental sciences

For a reference copy of the document with all sections, see nature.com/documents/nr-reporting-summary-flat.pdf

Life sciences study design

All studies must disclose on these points even when the disclosure is negative.

Sample size	This is not relevant to our study because we did not generate samples to calculate the statistics, we directly calculate the mean and SD from the probability matrix.
Data exclusions	To compare between datasets, we mapped all the proteins/genes to ORFs using Saccharomyces Genome Database and hORFeome Database9.1. Any proteins/genes that can not be mapped are excluded in our study. Besides, all self-interactions are excluded.
Replication	The analysis was replicated three times before submission with python 3.10.6 and python 3.11.0. All attempts at replication were successful.
Randomization	This is not relevant to our study because we did not allocate samples to experimental groups.
Blinding	This is not relevant to our study because we did not study the group allocation.

Reporting for specific materials, systems and methods

We require information from authors about some types of materials, experimental systems and methods used in many studies. Here, indicate whether each material, system or method listed is relevant to your study. If you are not sure if a list item applies to your research, read the appropriate section before selecting a response.

Materials & experimental systems

n/a	Involved in the study
☒	☐ Antibodies
☒	☐ Eukaryotic cell lines
☒	☐ Palaeontology and archaeology
☒	☐ Animals and other organisms
☒	☐ Clinical data
☒	☐ Dual use research of concern

Methods

n/a	Involved in the study
☒	☐ ChIP-seq
☒	☐ Flow cytometry
☒	☐ MRI-based neuroimaging
